# Lactic acid bacteria and yeasts involved in the fermentation of*amabere amaruranu*, a Kenyan fermented milk

**DOI:** 10.1002/fsn3.162

**Published:** 2014-08-21

**Authors:** Bitutu Nyambane, William M Thari, John Wangoh, Patrick M K Njage

**Affiliations:** 1 Food Technology Division, Kenya Industrial Research and Development InstitutePO Box 30650-00100, Nairobi; 2 Department of Food Science, Nutrition and Technology, University of NairobiPO Box 29053-00100, Nairobi

**Keywords:** *Amabere amaruranu*, container type, *Enterobacteriaceae*, identification, lactic acid bacteria, traditional fermentation, yeasts and molds

## Abstract

Indigenous fermented milk products contain microbiota composed of technologically important species and strains which are gradually getting lost with new technologies. We investigated the microbial diversity in*amabere amaruranu*, a traditionally fermented milk product from Kenya. Sixteen samples of the product from different containers were obtained. One hundred and twenty isolates of lactic acid bacteria (LAB) and 67 strains of yeasts were identified using API 50 CH and API 20 C AUX identification kits, respectively. The average pH of all the traditional fermented samples was 4.00 ± 0.93. Lactobacilli, yeasts, and molds as well as*Enterobacteriaceae* counts from the plastic containers were significantly higher (*P* < 0.05) than those from gourd.*Enterobacteriaceae* were below 1.00 ± 1.11 log_10_ cfu/mL in products from the gourds and 2.17 ± 1.92 log_10_ cfu/mL from the plastic containers. The LAB species were identified as*Streptococcus thermophilus* (25%),*Lactobacillus plantarum* (20%), and*Leuconostoc mesenteroides* (20%). The predominant yeasts were*Saccharomyces cerevisiae* (25%),*Trichosporum mucoides* (15%),*Candida famata* (10%), and*Candida albicans* (10%). The type of vessel used for fermentation had no significant influence on the type of isolated and identified species. The diverse mixture of LAB and yeasts microflora forms a potential consortium for further product innovation in*amabere amaruranu* and other fermented milk products.

## Introduction

Modern socio-economic changes mean that some traditional technologies for the production of fermented foods might eventually be lost together with the associated microorganisms (Akabanda et al. 2013). This underscores the importance of studying indigenous fermented products for their microbiota which might yield technologically important species and strains. Microorganisms present in traditionally fermented milk products have been documented in various studies (Gonfa et al. 1999; Beukes et al. 2001; Lore et al. 2005; El-Baradei et al. 2008; Mathara et al. 2008; Njage et al. 2011; Akabanda et al. 2013). The most predominant lactic acid bacterial (LAB) genera that were isolated from these products included*Lactobacillus fermentum* (Beukes et al. 2001),*Lactobacillus plantarum* (Mathara et al. 2008),*Leuconostoc mesenteroides* (Lore et al. 2005), and*Streptococcus thermophilus* (El-Baradei et al. 2008). The most commonly encountered yeast genera were*Saccharomyces*,*Candida,* and*Trichosporon* (Beukes et al. 2001; Lore et al. 2005; Njage et al. 2011).

*Amabere amaruranu* is a fermented milk product that is prepared by spontaneous fermentation of milk using a gourd made from the hollowed out fruit of*Lagenaria* spp*. Amabere amaruranu* is popular among members of Abagusii, who inhabit the Kisii highlands on the southwestern part of Kenya. It is made from cow's milk that is heated and held at boiling point for 10 min. The milk is then added to a small portion of fermented milk from a previous batch after cooling for 10–20 min and left to ferment at ambient temperature ranging from 10 to 32°C. Two types of containers are used for fermentation, gourd and plastic containers. Milk fermented using the gourd is more popular of the two. The product is white in color, has a grain-like appearance, low viscosity, is lumpy in nature, and acidic in taste (O. Arasa, personal communication). There is no substantial information available concerning the microbiological analysis of*amabere amaruranu*.

The objective of this study was to isolate, enumerate, and identify the dominant microorganisms in the fermented*amabere amaruranu*. Laboratory fermentation replicating the traditional fermentation was also carried out and monitored.

## Materials and Methods

### Sample collection

A total of 16 samples of traditionally prepared*amabere amaruranu* were collected from several randomly identified milk processors in the Kisii Region. Eight samples were obtained from the traditional gourd and eight of the samples were from plastic containers. The milk was collected in sterile bottles and transported to the Kenya Industrial Research and Development Institute (KIRDI) microbiology laboratories in a cool box and stored at 4–6°C before analysis. The samples were analyzed within 24 h of collection.

### Laboratory-based production of*amabere amaruranu*

*Amabere amaruranu* was prepared using the traditional method. Briefly, the milk was heated and held at boiling point for 10 min. The milk was then added to a small portion of fermented milk from a previous batch at 50°C and left to ferment at ambient temperature ranging from 18 to 32°C. A total of four laboratory produced samples were prepared, two using the gourds and the other two using the plastic containers, that had been collected from fermented milk processors and had previously been used for fermentation. The gourd that was used for fermentation was prepared by the traditional milk processors and cleaned using pebbles and hot water before they were handed over. The plastic containers were cleaned using hot water. In the laboratory, all the vessels were cleaned using boiling hot water and left to dry at ambient temperature. The pH and microbial counts (total viable counts, LAB,*Enterobacteriaceae*, and yeasts and molds) were determined after 0, 4, 8, 24, 48, 72, and 96 h. All the laboratory experiments were replicated twice.

### Enumeration of microorganisms

Twenty five milliliters of each sample was homogenized in 225 mL sterile diluent (0.1% bacteriological peptone (Himedia, Mumbai, India) and 0.85% NaCl (Loba Chemie, Mumbai, India), pH 7.0 using a stomacher (Stomacher- Bagmixer, Buch and Holm, Interscience, France) for 30 sec, at a preset speed. Ten-fold serial dilutions (10^−1^ to 10^−8^) were made with the same diluent and 1 mL was pour plated in duplicates on various media for enumeration of isolates as described by Harrigan and McCance (1998).Total viable counts were enumerated on pour plates of plate count agar (PCA) (Oxoid Ltd, Basingstoke, UK) and incubated at 30°C for 48 h. Mesophilic lactobacilli and Leuconostocs were enumerated on pour plates of de Man, Rogosa and Sharpe (MRS) agar (Oxoid) and incubated anaerobically at 35°C for 48 h, using the gas-generating kit anaerobic system (Oxoid). Lactococci were enumerated on M17 agar (Oxoid) followed by the anaerobic method and incubated at 35°C for 48 h. Yeasts were enumerated on potato dextrose agar (PDA) (Oxoid), acidified to pH 3.7 using 10% tartaric acid (Lobachemie), and incubated at 25°C for 5 days.*Enterobacteriaceae* were enumerated on pour plates of violet red bile glucose agar (VRBGA) (Oxoid), after incubation at 37°C for 24 h. Typical*Enterobacteriaceae* consisted of red to dark purple pin-point colonies surrounded by a dark purple halo on VRBGA.

### Isolation of microorganisms

Discrete colonies from pour plates of the highest dilution of each media were selected and isolated based on their shape, size, colour, and gloss. The isolated colonies were purified by repetitive streaking thrice on isolation media and stored in 0.25 mol/L sucrose solution at −18°C until required for identification. In addition, pure yeast cultures were also sub-cultured onto PDA slants, incubated at 25°C and stored at 4–6°C for further identification up to the genus and species level.

### Identification of lactic acid bacteria and yeasts

LAB strains were characterized according to methods recommended by Harrigan and McCance (1998). All strains were subjected to Gram staining, microscopic cell morphology, the catalase test, growth at 15°C and 45°C, and gas production from glucose and arginine hydrolysis. The API 50 CH (BioMérieux, Marcy l'Etoile, France) strips were used to identify the LAB isolates to the species level. Primary classification of yeast colonies from PDA plates was based on colony characteristics (color and shape), mode of vegetative propagation, formation of hyphae or pseudohyphae, and ascospore production. Identification of the yeast isolates to the species level was done using API 20 C AUX (BioMérieux). APILAB PLUS V3.2.2 software database was used for interpretation of the results.

### Statistical analysis

The data on microbial counts were first transformed by a logarithmic (log_10_) transformation before computing the mean log_10_ count and standard deviations. The independent*t*-test was used to determine whether or not a significant difference existed between the milk fermented using the gourd and that fermented using a plastic container, with respect to the pH and microbial counts. The level of significance was 5%. Statistical analysis was done using Microsoft Excel 2010.

## Results and Discussion

### Acidification of*amabere amaruranu*

There was no significant difference (*P *> 0.05) in the mean pH between samples from the two fermentation vessels. The mean pH of the samples obtained from gourd was 4.54 ± 0.88 while that of samples from plastic containers was 4.53 ± 1.05 after 96 h of fermentation.

### Enumeration of microorganisms associated with*amabere amaruranu*

Table1 shows total viable counts, LAB, yeasts and molds, and*Enterobacteriaceae* in the field samples. High total viable counts were observed in all the samples. There was no significant difference (*P *> 0.05) in the numbers of total viable microorganisms and the LAB between the samples from gourds and those from plastic containers.

**Table 1:** Microbiological counts of samples from traditionally fermented*amabere amaruranu*.

Microbial group	Log count (log_10_ cfu/mL)	
	Gourds (*n *= 8)	Plastic containers (*n *= 8)
Total viable counts	8.06 ± 0.59	8.24 ± 0.40
Lactobacilli	8.08 ± 0.53^1^	7.26 ± 0.59^1^
Lactoccoci	7.85 ± 0.67	7.12 ± 0.95
Yeasts and molds	4.65 ± 1.04^1^	6.07 ± 0.52^1^
Coliforms	0.59 ± 1.11^1^	2.17 ± 1.92^1^

1 Significant difference between samples from gourd and those from plastic containers.

However, the total viable counts, yeasts and molds as well as*Enterobacteriaceae* counts from the plastic containers were significantly higher (*P* < 0.05) than those from gourds. While*Enterobacteriaceae* were less than 1.00 ± 1.11 log_10_ cfu/mL in the samples from the gourds, they were present in those from plastic containers at 2.17 ± 1.92 log_10_ cfu/mL.

### Changes in microbial counts and pH during laboratory fermentation

**Figure 1:**
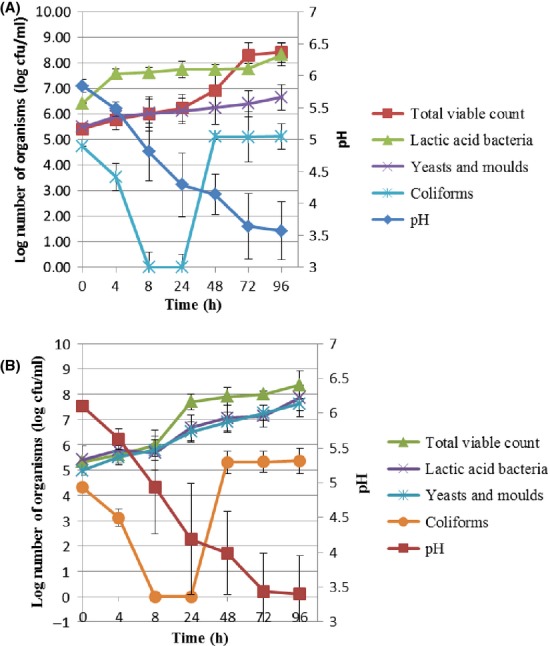
Changes on average in microbial numbers and pH during 96 h laboratory fermentation using (A) gourd and (B) the plastic containers.

The microbiological study of*amabere amaruranu* revealed the dominance of LAB in laboratory samples during the entire duration of fermentation, although yeasts were also present in considerably high numbers. Figure1 shows the changes on average in the microbial numbers and pH during laboratory fermentation using gourds and plastic vessels.

LAB counts increased from an initial level of 6.42 ± 0.01 in the gourd to 8.32 ± 0.45 log_10_ cfu/mL, while they increased from 5.41 ± 0.53 to 7.86 ± 0.50 log_10_ cfu/mL in the plastic container after 96 h of fermentation. The yeast population in both containers also increased with time from 5.50 at 0 h to 6.65 log_10_ cfu/mL at 96 h in the gourds and from 4.98 ± 0.19 at 0 h to 7.62 ± 0.50 log_10_ cfu/mL in the plastic vessels at 96 h. There was no significant difference (*P* > 0.05) in the LAB and yeast counts between the two fermentation vessel types after 96 h of fermentation.

The pH of the milk decreased from 5.84 ± 0.10 to 3.64 ± 0.45 in the gourd while it decreased from 6.10 ± 0.01 to 3.44 ± 0.55 in the plastic container after 96 h. The pH of the milk fermented in the plastic container decreased faster than that fermented using the gourd while milk fermented in the gourd emerged with a lower final coliform count than the plastic container. However, there was no significant difference between the two final counts (*P* > 0.05) after 96 h. The milk fermented in the gourd was expected to have a higher load of*Enterobacteriaceae*, yeasts, and LAB considering the difficulties experienced while cleaning it.

During the laboratory fermentation, the final LAB counts for the milk fermented using the gourd at 96 h were 8.32 ± 0.45 log_10_ cfu/mL, which reflected the growth of the LAB present in the backslope (8.3 ± 0.51 log_10_ cfu/mL). This was also observed in the plastic container where the LAB counts in the backslope (fermented milk from a previous batch) were 7.25 ± 0.50 log_10_ cfu/mL, and this increased to 7.86 ± 0.50 log_10_ cfu/mL in the final laboratory fermented milk.

The high numbers of LAB, accompanied by the low pH observed in samples obtained from the field and during the laboratory replication process (pH 3.64 for the gourd and pH 3.44 for the plastic) may be responsible for the sour taste, flavor and unique aroma of the product. The production of lactic acid gives the fermented product a sour taste and results in the formation of a coagulum (Narvhus and Gadaga 2003).

The LAB counts (10^−5^ to 10^−7^) are similar in range to other studies on traditionally fermented milk products. Lore et al. (2005) found counts ranging from 6.77 to 8.93 log_10_ cfu/mL in*suusac* while Mathara et al. (2008) found average values of 8.0 log_10_ cfu/mL in the study of*kule naoto*. Beukes et al. (2001) found mean counts ranging from 7.05 to 7.7 log_10_ cfu/mL in traditionally fermented milk in South Africa.

The yeast population increased steadily from 5.50 to 6.65 log_10_ cfu/mL in the gourd, while they increased from 6.65 to 7.62 log_10_ cfu/mL in the plastic container. The yeast counts recorded in this report were in similar range to those reported by Mathara et al. (2008) who found yeasts counts of <1.0–7.4 log_10_ cfu/mL. The presence of yeast in traditionally fermented milk products, in varying numbers, has been reported elsewhere (Beukes et al. 2001; Lore et al. 2005; Kebede et al. 2007; Njage et al. 2011). The frequent concurrence of LAB and yeasts has led to the suggestion that there could be interactions that may influence the product characteristics and quality (Narvhus and Gadaga 2003).

*Enterobacteriaceae* decreased from 4.73 and 4.32 log_10_ cfu/mL in the gourd and plastic container, respectively, to almost undetectable levels after 24 h, with a decrease in pH of both products to below 4.5. A decrease in pH occasioned by the production of organic acids in fermented milk products leads to inhibition of*Escherichia coli* and other coliforms (Gran et al. 2003).

However, after 48 h, there was an emergence of*Enterobacteriaceae* colonies, as identified by colony morphology, in both vessels and their population increased remarkably for the rest of the fermentation process. High prevalence of*Enterobacteriaceae* was also reported by Mathara et al. (2008) in the study of*kule naoto*. When the backslope method was used, acid-resistant strains of*E. coli* and coliforms may be present in the inoculum, which may explain the high numbers of*E. coli* and coliforms in the fermented product (Gran et al. 2003).

*Enterobacteriaceae* are normally associated with poor hygiene and their presence may be a pointer toward a potential health risk. Dirar (1993) observes that lack of pasteurization in traditionally fermented milk products is a major risk-enhancing factor. In our study, even though the milk is boiled for prolonged periods of time, this is insufficient to minimize the risk of contamination, coliforms were still detected, an indication of postheat treatment contamination.

It was observed that the plastic container began to accumulate high levels of gases and swell, and this coincided with the rapid increase in the numbers of the atypical coliforms. Atypical coliform colonies were not detected in the first 24 h. This could also be due to the presence of coliforms, such as*Enterobacter aerogenes* which produces a frothy product (Nout 1994).

### Identification of lactic acid bacteria

Table2 provides a summary of the phenotypic properties of LAB that were isolated from*amabere amaruranu* samples. All the isolates that were Gram positive, catalase negative, were rod or coccus shaped, and were considered to be LAB. They were further identified and shown to belong to three genera namely*Lactobacillus* (45%),*Streptococcus* (25%), and*Leuconostoc* (20%).

**Table 2:** Phenotypic properties of lactic acid bacteria from*amabere amaruranu*.

Property	Group						
	1	2	3	4	5	6	7
Cell morphology	Cocci	Cocci	Rods	Rods	Rods	Rods	Cocci/Rods
CO_2_ from glucose	−	+	−	−	−	+	V
NH_3_ from arginine	−	−	−	−	−	−	−
Growth at 15°C	−	+	+	−	−	−	V
Growth at 45°C	+	−	−	+	+	+	V
Substrate fermentation							
d-arabinose	−	−	−	−	−	+	a
Ribose	−	+	+	−	−	+	a
d-xylose	−	+	−	−	−	+	a
Galactose	−	+	+	−	+	+	a
Glucose	+	+	+	+	+	+	a
Fructose	+	+	+	+	+	+	a
Mannose	+	+	+	−	+	W	a
Rhamnose	−	−	−	−	−	−	a
Mannitol	−	−	+	−	−	−	a
Esculin	−	+	+	+	+	−	a
Salicin	−	−	+	−	−	−	a
Cellobiose	−	+	+	−	−	+	a
Maltose	+	+	+	−	+	+	a
Lactose	+	+	+	+	+	+	a
Melibiose	−	+	+	−	−	+	a
Sucrose	+	+	+	−	−	+	a
Trehalose	−	+	+	−	+	+	a
Melezitose	−	−	+	−	−	−	a
d-raffinose	+	+	+	−	−	+	a
2-keto-glucose	−	−	−	−	−	−	a
Identity	*Streptococcus thermophilus* (30 isolates)	*Leuconostoc mesenteroides* subsp.*Mesenteroides* (24 isolates)	*Lactobacillus plantarum* (24 isolates)	*Lactobacillus bulgaricus* (18 isolates)	*Lactobacillus helviticus* (6 isolates)	*Lactobacillus fermentum* (6 isolates)	Not identified (12 isolates)

+, positive reaction; −, negative reaction; w, weak reaction; v, variable reaction.

The most frequently isolated species comprising of 25% of all LAB was*S. thermophilus*, which was evenly distributed in samples from both containers. These organisms were characterized by the lens-shaped colonies growing on M17 agar and could not grow at 15°C but were able to grow at 45°C. Tests using API 50 CH galleries identified these strains at 90% level of certainty as*S. thermophilus*.

*Streptococcus thermophilus* has been reported to play a prominent role in the fermentation of dairy products. It was also reported as the most dominant species in the fermentation of*zabady* a fermented milk product from Egypt (El-Baradei et al. 2008) and ergo, a traditional fermented milk product from Ethiopia (Gonfa et al. 1999).

*Lactobacillus bulgaricus* subsp.*bulgaricus* made up 15% of the total number of lactic acid bacterial isolates.*Streptococcus thermophilus* in combination with*Lactobacillus delbrueckii* subsp.*bulgaricus* play a key role in the fermentation of yoghurt and need to be viable and abundant in the final product (CAC 1994).

*Leuconostoc mesenteroides* subsp.*Mesenteroides* consisted of 20% of all the isolates. It was also evenly distributed in samples from the gourd and plastic containers.*Leuconostoc mesenteroides* subsp*. mesenteroides* fermented most of the sugars present except d-Arabinose, rhamnose, mannitol, salicin, melezitose, and 2-keto-gluconate. It was similarly one of the most frequently isolated species in*suusac,* a traditional fermented camel milk product (Lore et al. 2005). It has also been isolated from*nunu* (Akabanda et al. 2013), a Ghanaian fermented milk product.*Leuconostoc* spp. are able to convert citrate into aroma compounds such as acetoin and diacetyl (Lore et al. 2005). This may indicate an important functional characteristic of this organism in*amabere amaruranu*.

Another highly prevalent*Lactobacillus* in*amabere amaruranu* was*L. plantarum,* which comprised 20% of the isolates. A total of 60% of the*L. plantarum* isolates were from the gourd while the rest were from plastic containers. Mathara et al. (2008) found that*L. plantarum* was the most dominant lactobacilli in*kule naoto*. It was also isolated by Beukes et al. (2001) from South African traditional fermented milk products and Lore et al. (2005) from*suusac*.*Lactobacillus plantarum* is commonly associated with plant-based fermentations (Holzapfel 1997) such as production of lactic acid in pickles and sauerkraut (Stiles and Holzapfel 1997). Their presence in fermented milk products could be due to the use of plant materials such as the gourd for fermentation and the adaptation of strains to milk (Mathara et al. 2008).*Lactobacillus plantarum* is also homofermentative, fermenting lactose to produce lactic acid as the main metabolic product. This could suggest that it also plays a significant role in lactic fermentation of*amabere amaruranu*.

A further 5% of the isolates were identified as*Lactobacillus helveticus* and were isolated from milk samples obtained from the plastic container.*Lactobacillus helveticus* has been associated with the production of bacteriocins that inhibit the growth of*Staphylococcus aureus*,*Salmonella species*, and*E. coli* (Stiles and Holzapfel 1997). It has also been isolated from*nunu* (Akabanda et al. 2013).

### Identification of yeasts

Table3 provides a summary of the phenotypic properties of the yeasts isolated from*amabere amaruranu* samples. All the yeast species isolated in this study were present in samples were obtained from both the gourds and the plastic containers. This shows uniformity in the microbial diversity in the milk fermented using both containers. No molds were detected.

**Table 3:** Phenotypic properties of yeasts isolated from*amabere amaruranu*.

Property	Yeast isolates				
	Group 1	Group 2	Group 3	Group 4	Group 5
Colony color	White	White	White	White	White
Ascospores	Present	Absent	Absent	Absent	Absent
Budding cells	Present	Present	Present	Absent	V
Hyphae/pseudohyphae	Present	Present	Absent	Present	V
Substrate fermentation					
Glucose	+	+	+	+	+
Glycerol	−	+	+	−	+
2-keto-d-gluconate	−	+	+	−	+
l-arabinose	−	−	+	−	+
d-Xylose	−	+	+	−	+
Adonitol	−	+	+	−	+
Xylitol	−	+	+	−	+
Galactose	+	+	+	+	+
Inositol	−	+	−	−	+
Sorbitol	−	+	+	−	+
α-methyl-d-glucoside	−	+	+	−	+
*N*-acetyl-glucosamine	−	+	+	+	+
Cellobiose	−	+	+	−	+
Lactose	−	+	+	−	+
Maltose	+	+	+	+	+
Sucrose	−	+	+	−	+
Trehalose	−	+	+	−	+
Melezitose	−	+	+	−	+
Raffinose	−	+	−	−	+
Identity	*Saccharomyces cerevisiae* (17 isolates)	*Trichospora mucoides* (10 isolates)	*Candida famata* (7 isolates)	*Candida albicans* (7 isolates)	Low discrimination (26 isolates)

+, positive reaction; −, negative reaction; v, variable reaction.

The yeast species that were identified belonged to the genera*Saccharomyces* (25%),*Candida* (20%), and*Trichosporon* (15%). Similar genera of yeasts isolated from*amabere amaruranu* were reported by Njage et al. (2011) who characterized the yeasts associated with fermented camel milk. The most frequently isolated species included*Saccharomyces cerevisiae* (25%:55% from the gourd and 45% from the plastic container),*Trichosporon mucoides* (15%:55% for the gourd and 45% from the plastic container),*Candida famata* (10%:50% from the gourd and 50% from the plastic vessel) and*Candida albicans* (10%:50% from the gourd and 50% from the plastic vessel). These isolates were unambiguously identified using the API software database.

In the present study,*S. cerevisiae* isolates were able to ferment galactose, sucrose, raffinose, and glucose but failed to ferment lactose, while they showed the presence of pseudohyphae. Nonlactose fermenting yeasts, such as*S. cerevisiae* utilize galactose that is mainly secreted by most of the lactobacilli (Hickey et al. 1986).

The API 20C AUX was unable to conclusively identify 40% of the yeast isolates and these were assigned presumptively to a number of yeast species at different identity levels. Four isolates showed similarities to*C. albicans* (78% identification percentage) and also*Candida krusei/inconspicua* (5.4% identification percentage) from the API software database. Three other isolates were identified as*Candida pelliculosa* (61.6% identification percentage) and*Candida sphaerica* (30.6% identification percentage). The remaining isolates could not be accurately identified using API kits and were found to be either*Cryptococcus laurentii* (46.1% identification percentage)*Candida humicolus* (29.5% identification percentage) or*T. mucoides* (23.4% identification percentage). Due to the limited capacity of API system to satisfactorily identify these strains, the use of molecular tools for more accurate typing of these organisms is recommended.

The presence of yeasts in traditionally fermented milk products has been reported by many investigators (Lore et al. 2005; Njage et al. 2011; Akabanda et al. 2013). They consist of lactose fermenting and nonlactose fermenting species (Gilliland 1990). The predominant yeast species encountered in*nunu* were*S. cerevisiae* and*Pichia kudriavzevii* (Akabanda et al. 2013), whereas*S. cerevisiae, T. mucoides, C. incospicua,* and*C. famata* were found in*suusac* (Njage et al. 2011). In the present study, the most dominant species was*S. cerevisiae*.

The source of yeast in traditionally fermented milk products could be contamination from the environment and also from the equipment associated with milking and processing equipment, especially the fermentation vessel (Narvhus and Gadaga 2003). In a study conducted by Kebede et al. (2007), the type of container used for fermentation did not necessarily influence the yeast counts, but had an effect on the diversity of yeasts isolated from the different containers used. They established that a clay pot gave a product of diverse flavors due to the many different microorganisms isolated.

*Saccharomyces cerevisiae* has been associated with the production of alcohols and other aroma compounds, stimulation of LAB, improvement of nutritional value, and inhibition of undesirable microorganisms (Jespersen 2003). However, the yeasts present in this product need to be investigated further to establish their exact role in the fermentation process, including their interaction with LAB and their metabolic properties.

The presence of*C. albicans* is of particular concern since it is rarely isolated from fermented milk products. It is mostly known as an opportunistic pathogen that can cause superficial, localized, and/or systemic infections in humans (Ryan 1990). Its presence in this product requires further investigations because its significant growth presents a safety concern.

The API 20C AUX was able to identify 60% of the isolates accurately while the remaining isolates were assigned two to three yeast species at different identity levels. Similar findings were reported by Njage et al. (2011). The fact that species that are found in milk might not be present in databases of commercial identification methods such as API kits could explain the limited capacity of the commercial kits to identify the yeast isolates accurately (Njage et al. 2011).

## Conclusions

Microorganisms involved in*amabere amaruranu* fermentation were found to consist of LAB from the three genera*Lactobacillus, Leuconostoc*, and*Streptococcus*. The most prevalent genus was*Lactobacillus* while the most dominant species were*S. thermophilus* and*L. mesenteroides* subsp.*Mesenteroides*. Yeasts included*Candida, Saccharomyces* and*Trichosporon*. The most predominant yeast species was*S. cerevisiae*. These microorganisms should be tested for their technological properties, microbial interactions and possible inhibitory effects against spoilage and pathogenic microorganisms. This will enable product development and innovation for a more predictable fermentation and quality using starter cultures of mixed LAB and yeast cultures.
